# Development of a smart pH-responsive nano-polymer drug, 2-methoxy-4-vinylphenol conjugate against the intestinal pathogen, *Vibrio cholerae*

**DOI:** 10.1038/s41598-023-28033-0

**Published:** 2023-01-23

**Authors:** Hema Bhagavathi Sarveswari, Krishna Kant Gupta, Ramyadevi Durai, Adline Princy Solomon

**Affiliations:** 1 Quorum Sensing Laboratory, Centre for Research in Infectious Diseases (CRID), School of Chemical and Biotechnology, SASTRA Deemed to be University, Thanjavur, 613401 India; 2 School of Chemical and Biotechnology, SASTRA Deemed to be University, Thanjavur, 613401 India; 3 Pharmaceutical Technology Laboratory, School of Chemical and Biotechnology, SASTRA Deemed to be University, Thanjavur, 613401 India

**Keywords:** Biofilms, Microbiology, Bacterial pathogenesis

## Abstract

*Vibrio cholerae* causes cholera, an acute diarrhoeal disease. The virulence in *V. cholerae* is regulated by the quorum-sensing mechanism and response regulator LuxO positively regulates the expression of virulence determinants adhesion, biofilm formation, and cholera toxin production. Previous in-silico studies revealed that 2-methoxy-4-vinylphenol could bind to the ATP binding site of LuxO and the complex was compact and stable in pHs like intestinal pHs. Here, we have explored the polymeric nano-formulation of 2-methoxy-4-vinylphenol using cellulose acetate phthalate for controlled drug release and their effectiveness in attenuating the expression of *V. cholerae* virulence. Physico-chemical characterization of the formulation showed particles with a mean size of 91.8 ± 14 nm diameter and surface charge of − 14.7 ± 0.07 mV. The uniform round polymeric nanoparticles formed displayed about 51% burst release of the drug at pH 7 by 3rd h, followed by a controlled linear release in alkaline pH. The polymeric nanoparticles demonstrated a tenfold increase in intestinal membrane permeability ex-vivo. At lower concentrations, the 2-methoxy-4-vinylphenol polymeric nanoparticles were non-cytotoxic to Int 407 cells. In-vitro analysis at pH 6, pH 7, pH 8, and pH 9 revealed that cellulose acetate phthalate—2-methoxy-4-vinylphenol nanoparticles were non-bactericidal at concentrations up to 500 μg/mL. At 31.25 μg/mL, the nanoparticles inhibited about 50% of the biofilm formation of *V. cholerae* MTCC 3905 and HYR14 strains. At this concentration, the adherence of *V. cholerae* MTCC 3905 and HYR14 to Int 407 cell lines were also significantly affected. Gene expression analysis revealed that the expression of *tcp*, *qrr*, and *ct* at pH 6, 7, 8, and 9 has reduced. The CAP-2M4VP nanoparticles have demonstrated the potential to effectively reduce the virulence of *V. cholerae* in-vitro.

## Introduction

Cholera is an acute diarrheal disease caused by the Gram-negative bacteria *Vibrio cholerae*. The pathogen inhabits aquatic reservoirs and only two serogroups O1 and O139 causes cholera*. V. cholerae* spreads by faecal-oral route through the ingestion of contaminated food and water^1^. The ingested bacteria could be either in the free-living state or in the form of biofilms^2^. With the aid of the acid and stress tolerance mechanisms, *V. cholerae* evades the hostile environment in the host and establishes infection in the small intestine. Cholera has a huge impact on public health, especially affecting underdeveloped or developing countries due to lack of clean water, poor sanitation, and poor hygiene^3^. In 2017, about 1,227,391 cholera cases and 5654 deaths were reported from 34 countries. The highest number of cases were reported from Yemen, of which 41% resulted in deaths^4^.

Between 2009 and 2017, about 559 cholera cases were reported across 27 states and union territories in India. The highest cases were noted in Karnataka, followed by West Bengal and Assam. Alarmingly, out of 62 antibiotic resistance outbreaks, 2/3rd of the cases were recorded in West Bengal, Maharashtra, Odisha, and Delhi^5^. Various measures have been implemented by health agencies across the world to control and prevent cholera. Anti-virulence agents that can be safely delivered, effective and affordable is necessary, as cholera high-risk populations are from the lower economic status^6^.

*Vibrio cholerae* virulence is regulated by the quorum sensing (QS) system. At low cell density, the QS signalling molecules (CAI-1, AI-2) activate the LuxO of *V. cholerae*, which in turn positively regulates the expression of virulence including biofilm and cholera toxin. This is achieved by the positive regulation of *qrr* 1–4 sRNAs and AphA. At the same time, the active LuxO represses HapR, the regulator of HA/protease gene *hap*. At high cell density (HCD), LuxO is inactive leading to the repression of biofilm and cholera toxin. The HapR is also derepressed that positively regulates HapA. This causes the *V. cholerae* to detach and disperse from the small intestine in the diarrhoea to the environment^7^.

To target *V. cholerae* at the site of its primary infection—the small intestine, the drug should be protected from metabolism and should reach the target site at effective bioavailable concentrations. Polymeric nanocarrier delivery systems allow controlled delivery of the drug at the targeted sites^8^. Usage of pH-responsive drug delivery systems has been effective in achieving stability of drugs in the stomach and providing controlled release in the small intestine^9^. One of the commercially available pH-sensitive polymers is cellulose acetate phthalate (CAP), pharmaceutically well-established for the enteric coating of capsules and tablets. CAP dissolves only at pH conditions > 5.8^10^. The CAP polymer has been reported to be successful in the development of vaccines through the delivery of antigens in the intestine to curb microbial infections^10^.

Previously, we have shown that the phenolic compound 2-methoxy-4-vinylphenol (2M4VP) present in the organic extract of *Micromonospora* sp. RMA46 could interact with the active site of LuxO of *V. cholerae* multi-drug-resistant strain HYR14^11^. In this study, polymeric nanoparticles of 2M4VP using CAP have been explored to develop a formulation that allows the release of the drug in response to the alkaline pH of the small intestine. This formulation was further evaluated for its ability to inhibit *V. cholerae* virulence and aimed to reduce the pathogen’s virulence.

## Materials and methods

### Bacterial strains, culture condition, and materials

*Vibrio cholerae* strains MTCC 3905 (reference strain), and *Vibrio cholera* biofilm-forming rugose strain, HYR14 obtained from MTCC, Chandigarh and NICED, India, respectively were used throughout this study^12^. The HYR14 strain was gifted by Prof. Thandavaraya Ramamurthy, National Institute of Cholera and Enteric Diseases, Kolkata, India*.* The strains was cultured in thiosulfate–citrate–bile salts–sucrose (TCBS) agar to confirm cell viability and propagated under standard growth conditions in Luria–Bertani (LB) broth at 37 °C. An inoculum size of 0.2 (OD_595_) ≈ 10^5^ cells was used for all the experiments^13^.

Cellulose acetate phthalate (MW 2534.12) was purchased from SRL, India. Sodium hydroxide pellets (MW 40 g/mol), potassium dihydrogen phosphate (MW 136.09 g/mol), and boric acid (MW 61.83 g/mol) were purchased from Merck Life Science, India. Pluronic F-127 was purchased from Sigma-Aldrich. Acetone was purchased from Pure Chemicals Co. India and Methanol from HiMedia, India. Minimal Essential Medium (MEM) with Earle salts, l-Glutamine, NEAA and sodium bicarbonate, Phosphate buffered saline (pH 7), trypsin, fetal bovine serum, antibiotic antimycotic solution (100× with 10,000 U Penicillin, 10 mg Streptomycin and 25 µg Amphotericin B per ml in 0.9% normal saline) was purchased from HiMedia, India. MTT [3-(4,5-dimethylthiazole-2-yl)-2,5-diphenyl tetrazolium bromide] for cell proliferation assay was purchased from HiMedia, Mumbai, India. Int 407 cell lines were procured from National Centre for Cell Science (NCCS), Pune, India. Bacteriological isolation media, growth media, and Giemsa stain were procured from HiMedia, India. Int 407 cell lines were grown using MEM with 10% FBS and 1% antibiotic and antimycotic solution at 37 °C in a 5% CO_2_ incubator.

### Methods

#### Nanoparticles synthesis and lyophilization

The nanosuspension of 2-methoxy-4-vinylphenol (2M4VP) with cellulose acetate phthalate (CAP) was prepared by a modified nanoprecipitation method. About 10 mg of CAP was dissolved in 1 mL of acetone and 10 mg of 2M4VP in 1 mL of methanol. Both the drug and the polymer preparation were mixed to obtain the organic phase. About 0.5% of Pluronic® F127 in distilled water was prepared to form the aqueous phase. The 2 mL of the organic phase was added dropwise into the aqueous media using a syringe and the dispersion was left under constant and continuous stirring at 700 RPM at room temperature until the organic phase completely evaporates. The colloidal aqueous solution containing the nano-precipitated particles of CAP-2M4VP was then freeze-dried by lyophilization^14^ (Supplementary Fig. 1).

### Material characterization

The particle size distribution and stability of the CAP-2M4VP formulation were measured by the dynamic light scattering (DLS) and zeta potential using Zetasizer (NanoZs, Malvern Instruments, UK). The chemical interaction of drug and polymer present in the CAP-2M4VP particles was analyzed by Fourier-transform infrared spectroscopy (FTIR). The freeze-dried samples were placed on a hole of 1 mm on a stell gasket of 0.05 mm thickness on top of a diamond anvil cell. Then the sample was transferred to a stub and scanned from wavelength 4000 cm^−1^ to 400 cm^−1^^15^. The diffraction pattern of the CAP-2M4VP and 2M4VP were compared by X-ray diffractometer with Cu-Kα radiation at 30 mÅ of current and 40 kV volatage (Rigaku, Japan). The analysis was performed by scanning the samples at 2θ from 0° to 100°^16^. The variation in the thermal properties of the polymeric nanoparticles compared to the pure drug was studied using Thermogravimetry and Differential Scanning Calorimetry (NETZCH, Germany). About 5–10 mg of the samples were individually placed in an aluminum pan and heated at the rate of 10 °C/min up to 1000 °C and the result was represented as weight loss and heat flow against temperature. The surface morphology of the lyophilized CAP-2M4VP nanoparticles preparation and a pure sample of 2M4VP was captured after gold sputter-coating using Scanning Electron Microscope (VEGA3, TESCAN Analytics, Czech Republic).

### Entrapment efficiency

The percentage of 2M4VP entrapped within the nanoparticles was studied by centrifuging the CAP-2M4VP nanosuspension preparation at 12,000 rpm at 4°C for 20 min. The total amount of 2M4VP entrapped within the CAP nanoparticles was determined by the estimation of unentrapped free drug present in the supernatant. The absorbance of the supernatant solution was measured at 268 nm using respective blank. The percentage of entrapment efficiency was calculated by the following formula^17^,

$${\text{Percentage entrapment efficiency}} = \frac{{{\text{Total amount of drug }} - {\text{ Un-entrapped drug}}}}{{\text{The total amount of drug}}} \times 100.$$

### Drug content

The total amount of 2M4VP present within the CAP nanoparticles suspension was analyzed. About 1 mL of the nanosuspension was dissolved in 9 mL of methanol and left for 24 h at 4 °C. The solution was sonicated and the amount of drug present was measured using a UV–Visible spectrophotometer at 268 nm using a respective blank^18,19^.

### In-vitro drug release studies

The percentage of drug release of 2M4VP encapsulated in CAP nanosuspension was studied in the presence of dissolution media—buffers at various pHs (1.2, 5.8, 7, 8 and 9). About 1 mg/mL of the CAP-2M4VP nanoparticles were loaded into a dialysis membrane (HiMedia, Mumbai, India; Pore size: up to 14,000 Daltons) and the ends were sealed. The membrane was then placed inside a screw cap bottle filled with 5 mL of dissolution media of specific pH. The set-up was placed under constant stirring at 200 RPM at 37 °C. At regular time intervals, 5 mL of samples were removed using a syringe and were replaced with the 5 mL of the respective fresh buffers. The samples collected were analyzed at 268 nm using a UV–Visible spectrophotometer using respective blank. The study was performed in triplicate and presented as the mean with standard deviation limits^19^.

### Drug release kinetics

The cumulative percentage of drug release profiles obtained for pH 1.2, 5.8, 7.0, 8.0, and 9.0 was further analyzed by fitting the data into drug release kinetics models. The data from in-vitro drug release was fitted into zero-order, first-order, Higuchi, Korsmeyer–Peppas, Hixson–Crowell, Hopfenberg, Baker–Lonsdale, Weibull, and Gompertz kinetic models. The values of R^2^, Rate Constant (K), Sum of Squared Residual (SSR) and Release exponent (n) were identified for CAP-2M4VP nanoparticles at each pH included in this study^19^.

### Ex-vivo permeation assay

The permeation of CAP-2M4VP nanoparticles across excised goat intestinal mucosal membrane ex-vivo was performed using the Franz cell diffusion chamber. The Franz cell diffusion chamber comprises two compartments—an upper donor connected to a lower acceptor (receptor) compartment. The capacity of the acceptor compartment is approximately 15 mL and the available diffusion area is 1.8 cm^2^ in diameter. The acceptor compartment was filled with phosphate buffer pH 7 and a magnetic bead was placed inside. The 5 × 5 cm goat intestinal mucosal tissue was mounted on top of the acceptor compartment with the mucosal region facing towards the donor compartment with constant contact with the phosphate buffer pH 7 so that bubbles formation was prevented. The donor compartment was then placed on top of the intestinal tissue and both the compartment were held tightly in position with rubber clamps. The donor compartment was filled with phosphate buffer pH 7 containing 1 mg/mL of the CAP-2M4VP nanosuspension preparation. The opening of the donor compartment was covered with aluminium foil to prevent evaporation. The entire setup was placed on a magnetic stirrer at 50 RPM and continuously stirred at room temperature. About 500 μL of samples were collected from the receptor compartment at every 30 min interval for the first 2 h and then at 1 h interval for 8 h continuously. The cumulative amount of drug permeation was determined by a UV–Visible spectrophotometer at 268 nm. The volume withdrawn from the receptor compartment was replaced by the aliquoting same volume of the phosphate buffer into the donor compartment. The permeation data analysis was performed as per described by Ramyadevi and Rajan^19^. The results presented a mean ± SD of experiments performed in triplicates.

### Cell viability assay

The effect of the CAP-2M4VP nanoparticles on host cell viability was assayed using Int 407 cell lines, cultured in minimal essential medium (MEM) supplemented with 10% fetal bovine serum (FBS), 1% penicillin/streptomycin (w/v), and 1% l-glutamine using the MTT (3-(4,5-dimethylthiazol-2-yl)-2,5-diphenyltetrazolium bromide) assay. The Int 407 cell lines were procured from National Centre for Cell Science (NCCS), Pune, India. About 1 × 10^4^ Int 407 cells/well were seeded in a 96-well microtiter plate and incubated at 37 °C in a CO_2_ incubator for 24 h. After the incubation period, the cells were treated with various concentrations (100 μg/mL, 50 μg/mL, 25 μg/mL, 12.5 μg/mL, 6.25 μg/mL) of CAP-2M4VP and incubated for another 24 h at 37 °C in a CO_2_ incubator. After 24 h, 1 mg/mL of MTT was added, and the microtiter plate was incubated at 37 °C for 3 h. Dimethyl sulfoxide (DMSO) was added to dissolve the formazan crystals, and absorbance was read at 570 nm using a microplate reader spectrophotometer (Synergy H1)^20^.

### Inhibition of growth

The effect of the CAP-2M4VP nanoparticles on the growth of *V. cholerae* MTCC 3905 and HYR14 was evaluated using a 96-well microtitre plate assay from concentrations ranging from 500 to 0.06 μg/mL. *V. cholerae* MTCC 3905 and HYR14 strains were grown overnight in Luria–Bertani broth at 37 °C with shaking at 150 RPM. Briefly, overnight cultures of both the strains were diluted to 1:100 (OD_595_ = 0.2) using the fresh LB media. 100 μL of LB broth containing various concentrations of CAP-2M4VP from 500 to 0.06 μg/mL was aliquoted in the microtitre plate wells. To this 10 μL of diluted overnight cultures of *V. cholerae* were added. The plates were incubated at 37 °C for 24 h statically. After 24 h, the growth was measured by reading the absorbance at 595 nm using a microtitre plate reader (iMark, BIORAD, Japan). Experiments were performed in triplicates and the data are reported as mean with standard deviation^21^.

### Inhibition of biofilm formation

The ability of CAP-2M4VP to inhibit the biofilm formation of *V. cholerae* was evaluated by crystal violet assay using a 96-well microtitre plate. Briefly, overnight cultures of *V. cholerae* were diluted to 1:100 (OD_595_ = 0.2) using the fresh LB media. 100 μL of LB broth containing various concentrations of CAP-2M4VP from 250, 125, 62.50, 31.25, 15.63 to 7.81 μg/mL were aliquoted into the wells. To these 10 μL of diluted overnight cultures of *V. cholerae* strains MTCC 3905 and HYR14 were added. The wells without CAP-2M4VP served as control and wells without inoculum served as blank. The plates were incubated at 37 °C for 24 h statically. Biofilm quantification was performed using crystal violet assay as described. After 24 h, the planktonic *V. cholerae* cells were removed by washing with PBS thrice. To the wells, 150 μL of 0.2% crystal violet stain was added and incubated for 20 min for staining the adherent biofilm. After the incubation period, the unbound crystal violet stain was removed by washing the wells gently with PBS and the plates were air-dried. To this, 33% acetic acid was aliquoted to the wells to elute the crystal violet bound to the biofilm. The absorbance was read at 595 nm using a microtiter plate reader (iMark, BIORAD, Japan). Experiments were performed in triplicates and the data are reported as mean with standard deviation. The lowest concentration that inhibited about 50% of *V. cholerae* biofilm formation at all the pHs (pH 6, 7, 8, and 9) was considered a minimum biofilm inhibitory concentration of 50 (MBIC_50_)^22^.

### Fluorescent microscopy of biofilm

The inhibition of biofilm formation by CAP-2M4VP against *V. cholerae* MTCC 3905 and HYR14 was observed using fluorescent microscopy. About 25 mL of LB media adjusted to pH 6, pH 7, pH 8, and pH 9 containing 31.25 μg/mL of CAP-2M4VP were aliquoted to 50 mL sterile screw capped tubes. One percent of the diluted (1:100 (OD_595_ = 0.2)) overnight culture of *V. cholerae* strains MTCC 3905 and HYR14 were added to the tubes. Sterile glass slides were placed inside the tubes and incubated at static conditions for 24 h at 37 °C. pH-adjusted media tubes without CAP-2M4VP served as control. After the incubation period, the slides were recovered from the tubes and rinsed with sterile 0.9% NaCl to remove planktonic cells and media. As per the manufacturer’s protocol, the adherent biofilm was stained with BacLight Bacterial Viability Kit (ThermoFisher Scientific, USA). The images of biofilm were observed under a 40× objective using a fluorescent microscope (Nikon Eclipse Ts2)^23^.

### Cell adhesion assay

Int 407 cell lines were grown up to 90% confluency using MEM supplemented with 10% FBS without antibiotics at 37 °C in a 5% CO_2_ incubator. The cells were washed with phosphate-buffered saline at pH 7 and fresh media was added. The overnight culture of *V. cholerae* MTCC 3905 and HYR14 strains at a concentration of multiplicity of infection of 50 (MOI 50) was added along with 31.25 μg/mL of CAP-2M4VP. The plates were incubated at 37 °C in 5% CO_2_ for 1 h. After the incubation period, the cells were washed with PBS to remove the non-adherent bacteria. The cells were lysed using 0.1% of Triton X-100 prepared in PBS. The CFU of the adherent bacteria was enumerated and presented as a percentage of bacteria adherent to Int 407 cell lines. The experiments were performed in triplicates^24^. For imaging using light microscopy, the Int 407 cells were grown in Petri plates with sterile coverslips in MEM medium containing 10% FBS without antibiotics at 37 °C in a 5% CO_2_ atmosphere. The infection procedure was followed and after the infection period, the coverslips were removed and fixed with methanol for 30 min followed by staining with Giemsa stain for 20 min. The stained cells were washed with PBS and air-dried. The bacteria adherent to Int 407 cells were observed at 40× magnification using a microscope^25^.

### Gene expression analysis by quantitative RT-PCR

Gene expression analysis to understand the effect of CAP-2M4VP on the *V. cholerae* MTCC 3905 cells was performed using quantitative real-time PCR. RNA was extracted from 24 h culture of *V. cholerae* MTCC 3905 cells treated with 31.25 μg/mL of CAP-2M4VP following the manufacturer’s guidelines of the HiMedia RNA Extraction kit (MB613) (HiMedia, India). The purity and integrity of the isolated RNA were analyzed using NanoDrop (Thermo Fisher Scientific, United States). The conversion of total RNA to cDNA was performed using the iScript™ cDNA Synthesis kit following the manufacturer’s guidelines. The effect of CAP-2M4VP on the gene expression in MTCC 3905 was investigated using qRT-PCR using Real-Time PCR System (Applied Biosystems, USA). The primers used are described in Supplementary Table 1. 16S rRNA served as reference gene and the expression of genes was calculated using the 2^–ΔΔ^ ^CT^ method. The PCR conditions set were as described previously^26^.

### Statistical analysis

GraphPad Prism software version 8.0.2 (GraphPad Software Inc., San Diego, CA, United States) was used for statistical analysis of the results. The results were expressed as mean ± standard deviations (SD) and all the experiments were performed in triplicates. The significance was analyzed by One-way ANOVA followed by Dunnett’s multiple comparison test and Student *t*-test with *p* set at *p* ≤ 0.05.

## Results

### Nanoparticles characterization

**Figure 1:**
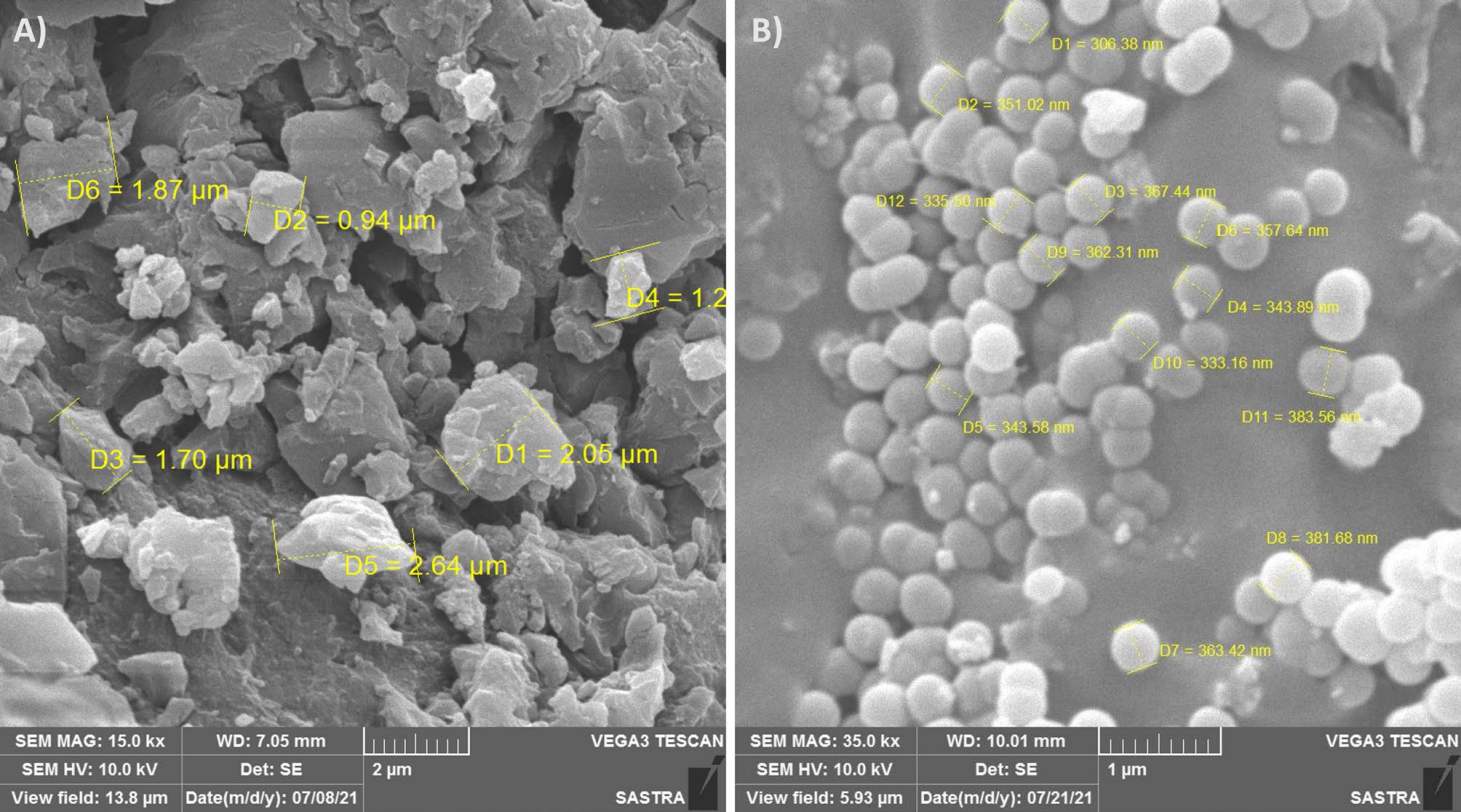
Scanning electron microscopy images showing (**A**) irregular flakes-like structures of pure sample of 2-methoxy-4-vinylphenol (2M4VP) at 15.0 kx magnification, (**B**) uniform spherical-shaped cellulose acetate phthalate nano-formulation of 2M4VP (CAP-2M4VP) at 35.0 kx magnification.

The physicochemical characterization of CAP-2M4VP nanoparticles at neutral pH revealed that the particles had a mean size of 91.8 ± 14 nm in diameter. The polydispersity index (PDI) was 0.251 ± 0.07, and the zeta potential is − 14.7 ± 0.07 mV indicating that the nanoparticles have good size distribution and are negatively charged (Supplementary Fig. 2a,b). Scanning electron microscopy images revealed that the pure drug (2M4VP) has an irregular flakes-like structure and the size ranged from 0.9 to 2.6 μM in size (Fig. 1a). The optimized formulation of CAP-2M4VP nanoparticles was smooth with uniform spherical structures. The size of the nanoparticles ranged between ~ 300 and ~ 400 nm (Fig. 1b). The total drug content in the optimized CAP-2M4VP nanosuspension was about 79.52 ± 0.2% and the highest entrapment efficiency of the nanoparticles was 59.7 ± 2%.

### Interaction between the polymer CAP and the drug 2M4VP

**Figure 2:**
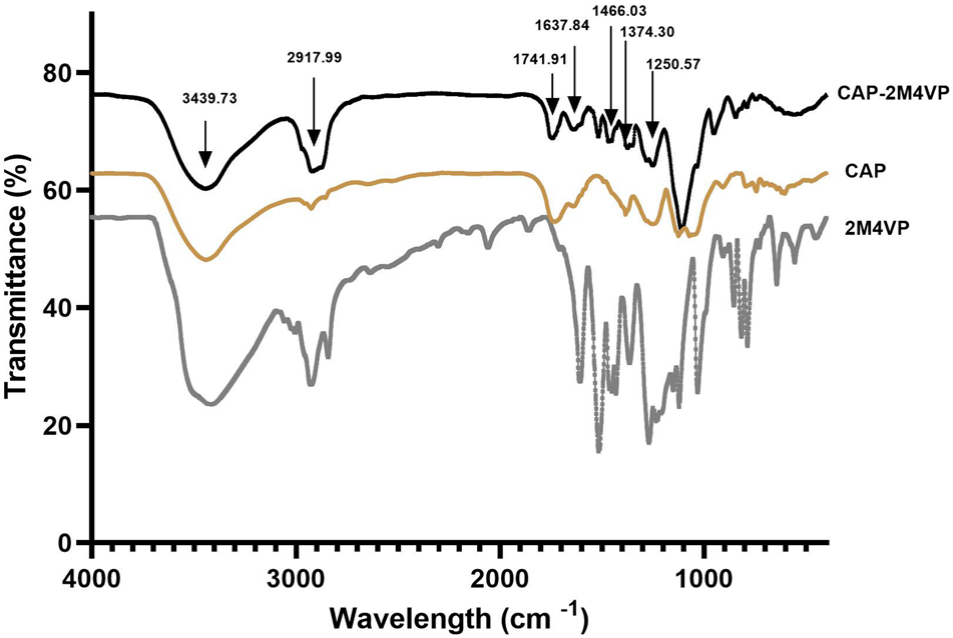
Comparison of Fourier-transform infrared (FTIR) spectra of pure sample 2-methoxy-4-vinylphenol (2M4VP), cellulose acetate phthalate (CAP) polymer and the cellulose acetate phthalate nano-formulation of 2M4VP (CAP-2M4VP) analysed by KBr pellet technique.

The FTIR spectrum of pure compound 2-methoxy-4-vinylphenol had displayed the characteristic peaks corresponding to its functional groups viz., at 3416 cm^−1^ for –OH stretching of alcohol, 2927 cm^−1^ for –C–H stretching and 1606 cm^−1^ for –C=C stretching of alkene double bond, 1450 cm^−1^ at –C–H bending of a methyl group, 1269 cm^−1^ and 1230 cm^−1^ for –C–O stretching and 1364 cm^−1^ for –OH bending. Therefore, the purity and chemical nature of the compound were confirmed (*NIST Chemistry WebBook, SRD 69, WILEY SpectraBase™*). In the case of the polymer Cellulose Acetate Phthalate, the peaks were obtained at 3439 cm^−1^ corresponding to –OH stretching of the alcoholic group, 2925 cm^−1^ for –C–H stretching of aliphatic chain, 1728 cm^−1^ for –C=O stretching, 1249 cm^−1^ for –C–O stretching, 1384 cm^−1^ for –OH bending and 742 cm^−1^ for –C=C bending frequency. The specific peaks at the defined wavenumbers represented the characteristic identity and chemical nature of the polymer material. The formulated nanoparticles of 2M4VP using CAP polymer had shown peaks at 3439 cm^−1^ representing –OH stretching of the alcoholic group, 2917 cm^−1^ for –C–H stretching, 1741 cm^−1^ for –C=O stretching, 1637 cm^−1^ for –C=C stretching, 1250 cm^−1^ for –C=O stretching, 1466 cm^−1^ for –C–H bending, and 1374 cm^−1^ for –OH bending (Fig. 2). The characteristic peaks of both drug and polymer molecules were displayed by the nanoparticles, wherein the functional groups and features of the drug were predominant without significant alterations in the chemical nature and stability/purity of the active compound.

### Crystal behaviour changes of 2M4VP within CAP

**Figure 3:**
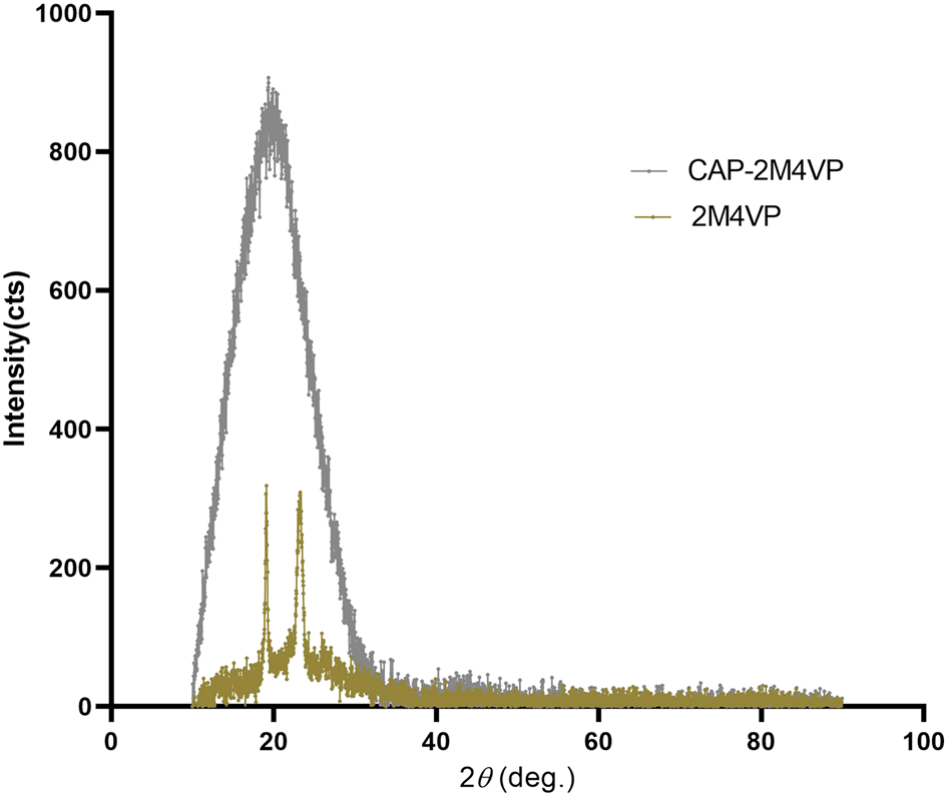
XRD pattern of pure sample 2-methoxy-4-vinylphenol (2M4VP) (gold line) compared to the cellulose acetate phthalate nano-formulation of 2M4VP (CAP-2M4VP) (grey line) indicating the transition from crystalline (2M4VP) to the amorphous nature (CAP-2M4VP) during the polymeric nano-particles formation.

The XRD pattern of the pure 2M4VP displayed sharp peaks with two high-intensity points between the range of 20–30 in the 2-theta scale, representing the characteristic crystalline nature of the drug. In the case of the XRD spectrum of 2M4VP loaded CAP nanoparticles, the crystalline peaks of the drug were not visualized due to encapsulation of 2M4VP in the CAP polymer matrix. This solid-state conversion of the drug in addition to amorphous CAP polymer confirmed the stability and entrapment of drug in the nanoparticles^27^ (Fig. 3). The transition of crystalline to amorphous nature in nanoparticle formulation could support uniform distribution, enhanced dissolution, and uptake of the drug in-vivo.

### Increased thermal stability of 2M4VP encapsulated within the polymer CAP

**Figure 4:**
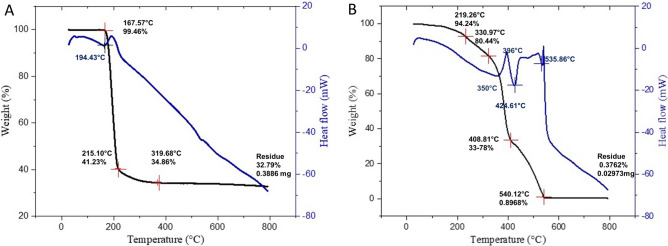
Thermal gravimetry–differential scanning calorimetry analysis of (**A**) pure sample of 2-methoxy-4-vinylphenol (2M4VP) and (**B**) cellulose acetate phthalate nano-formulation of 2M4VP (CAP-2M4VP) showing greater thermal stability of the polymeric nanoparticles than the pure drug 2M4VP.

The DSC thermogram of a pure sample of 2M4VP showed an endothermic peak at the temperature range of 194.43 °C, measured as the melting point of the crude sample, which could be confirmed by the corresponding sudden weight loss from 100 to 40% in the TGA curve. Further increase in temperature led to decomposition of the sample with a residual weight of 32% (Fig. 4a). In the case of CAP-2M4VP nanoparticles, the endothermic peak of the drug sample was diminished by the presence of amorphous CAP polymer, which exhibited thermal stability up to 300 °C. The DSC thermogram of the nanoparticles exhibited a shallow endothermic curve up to 350 °C, followed by a sudden exothermic hump at 396 °C. A corresponding stepwise non-linear thermogravimetry pattern was observed in the TG curve, representing the slow thermal degradation of the matrix nanoparticles from 0 to 550 °C, reaching the final residual weight of 0.89% (Fig. 4b). The results confirmed the formation of stable amorphous nanoparticles of 2M4VP uniformly dispersed within CAP polymer.

### In-vitro controlled release of 2M4VP from CAP

**Figure 5:**
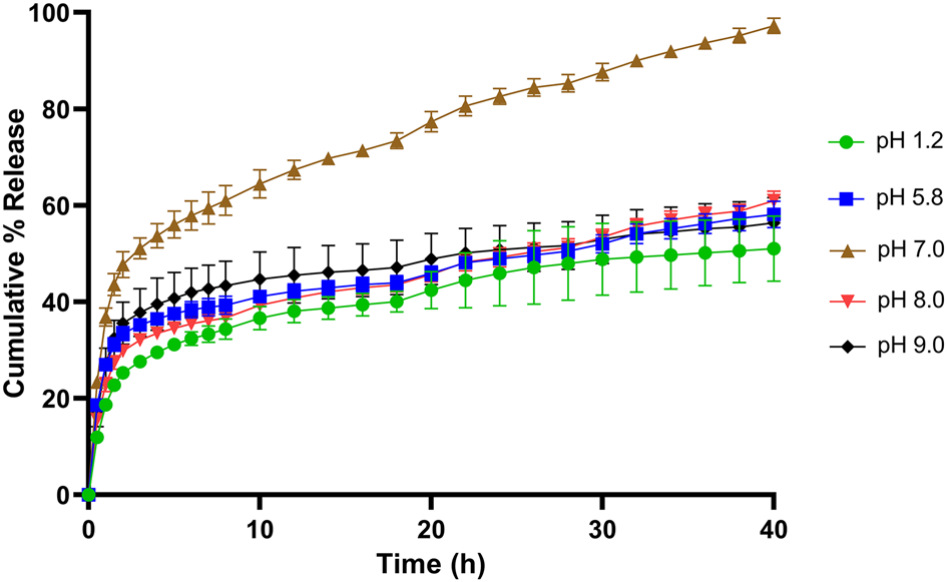
In-vitro drug release study of polymeric nano-formulation CAP-2M4VP in 0.1 N HCl pH 1.2 represented in green, phosphate buffer pH 5.8 represented in blue, phosphate buffer pH 7.0 represented in brown, phosphate buffer pH 8.0 represented in red and phosphate buffer pH 9.0 represented in black. The figure shows an initial burst release (51%) of 2M4VP from the CAP nanoparticles in pH 7 at 3rd h, followed by linear sustained release to reach 97% at 40th h. In pH 1.2, pH 5.8, pH 8.0 and pH 9.0 media, the cumulative percentage release of 2M4VP from the nanoparticles was 50% to 60% at the end of 40th h.

The release of 2M4VP from CAP at various pHs including pH 1.2, pH 5.8, pH 7.0, pH 8.0, and pH 9.0 was studied. At the end of the 3rd h, about 51% of drug release was noted in pH 7.0 while in pH 1.2, pH 5.8, pH 8.0, and pH 9.0 the release was about 30%. This showed an initial burst of drug release from CAP nanoparticles at pH 7.0. However, at pH 7.0 from 3rd h to 12th h, the release was slow and steady reaching about 67% drug release. During this period, the drug release was 38.06%, 42.24%, 40.82% and 45.52% at pH 1.2, pH 5.8, pH 8.0 and pH 9.0, respectively. At 24 h a maximum drug release of 82.62% was noted at pH 7.0, whereas only ~ 45% to 50% drug release was noted in other pHs included in this study. It took another 16 h (40 h) to reach the cumulative value of 97.24% of drug release at pH 7.0. At the end of this period (40 h), only 51.06%, 58.16%, 60.99%, and 56.39% release of drug in-vitro at pH 1.2, pH 5.8, pH 8.0, and pH 9.0 was noted, respectively. Overall, a burst release of drug (51%) in pH 7.0 at 3rd h followed by a linear sustained release to reach the maximum of 97% of drug till 40th h was observed. In pH 1.2, pH 5.8, pH 8.0, and pH 9.0 media, the drug release was slow and continuous reaching about 50% to 60% drug release only, at end of 40 h (Fig. 5).

### Mechanism of 2M4VP release from CAP

The cumulative drug release percentage data obtained from in-vitro drug release studies were analyzed for the rate of release and mechanism using kinetic models including zero order, first order, Higuchi, Korsmeyer–Peppas, Hixson–Crowell, Hopfenberg, Baker–Lonsdale, Weibull and Gompertz. The mechanism of diffusion of the drug from the nanoparticles was confirmed by Korsemeyer–Peppas and Weibull models, where the plots were linear with R^2^ values ranging between 0.97 and 0.99 at all pHs. The best fit model was the Korsmeyer–Peppas model, as the R^2^ values were greater than 0.97 at all pHs. This indicated that the mechanism of drug release was diffusion controlled. The n-value less than 0.20 explained the Fickian transport kinetics for the diffusion of the drug from nanoparticles. The Weibull kinetics model confirmed the drug release pattern based on the matrix type of systems, herein the 2M4VP loaded CAP matrix nanoparticles (Supplementary Table 2).

### Ex-vivo permeation of CAP-2M4VP

**Figure 6:**
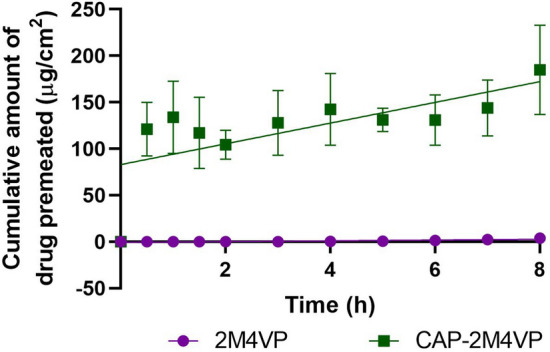
Ex-vivo permeation for the aqueous dispersion of pure drug 2-methoxy-4-vinylphenol (2M4VP) represented in purple colour and the cellulose acetate phthalate nano-formulation of 2M4VP (CAP-2M4VP) represented in green colour across the intestinal mucosal membrane using phosphate buffer pH 7. The figure shows tenfold increase in the cumulative amount of permeation of drug from the CAP-2M4VP nanoparticles compared to the pure sample of 2M4VP.

The formulated nanoparticles CAP-2M4VP showed about 119.0713 µg/cm^2^ permeation through the intestinal mucosal membrane within 2 h. Further, there was a linear increase from 3 h to 8 h to reach the cumulative permeation amount of 184.8705 ± 47.93 µg/cm^2^. The nano-formulation showed a steady-state flux of 11.15 ± 4.64 µg/cm^2^/h and permeability coefficient of 0.0111 ± 0.004 cm/h × 10^–3^ across the goat intestinal membrane (Fig. 6). Meanwhile, for the aqueous dispersion of pure 2M4VP, only 18.48 ± 6.67 µg/cm^2^ cumulative amount of drug was permeated across the intestinal mucosal membrane at the end of the 8th h. When compared to the pure 2M4VP permeation across intestinal membrane, the CAP-2M4VP formulation exhibited about a tenfold increase in intestinal membrane permeation. This phenomenon could be attributed to the enhanced permeation of nano-sized particles of CAP-2M4VP across the intestinal membrane in phosphate buffer pH 7^1^.

### CAP-2M4VP is non-cytotoxic at lower concentrations

**Figure 7:**
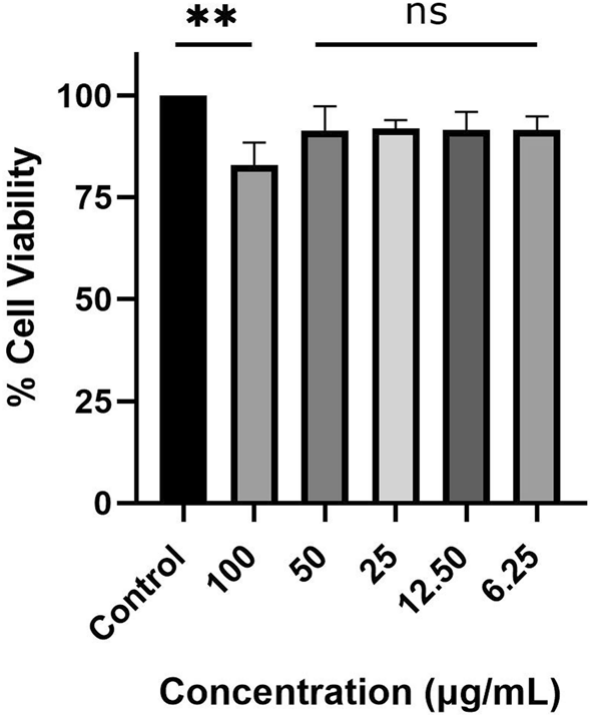
Percentage viability of Int 407 cell lines after exposure to various concentrations of CAP-2M4VP (100 μg/mL, 50 μg/mL, 25 μg/mL, 12.50 μg/mL, and 6.25 μg/mL) for 24 h at 37 °C. One-way ANOVA followed by Dunnett’s test for multiple comparisons was performed. **Indicates significant difference (**p < 0.01) compared to the untreated and ns denotes no significant difference. The figure shows significant difference in the viability of Int 407 cell lines when treated with CAP-2M4VP nano-formulation at the concentration of 100 μg/mL compared to untreated Int 407 cell lines (control). At the concentrations of 50 μg/mL, 25 μg/mL, 12.50 μg/mL, and 6.25 μg/mL, the treatment of CAP-2M4VP resulted in no significant difference in the viability of Int 407 cells compared to the untreated Int 407 cell lines (control).

The effect of the CAP-2M4VP formulation on the viability of Int 407 cells was investigated by MTT assay. The data shows that at lower concentrations ranging from 50 to 6.25 μg/mL the polymeric nano-formulation did not affect the viability of the Int 407 cell lines. At 100 μg/mL, the Int 407 cell viability was significantly affected (Fig. 7). This indicated that the polymeric CAP nano-formulation of 2M4VP is non-cytotoxic at a lower concentration.

### CAP-2M4VP inhibits biofilm formation of *V. cholerae* and is non-bactericidal at higher concentrations

**Figure 8:**
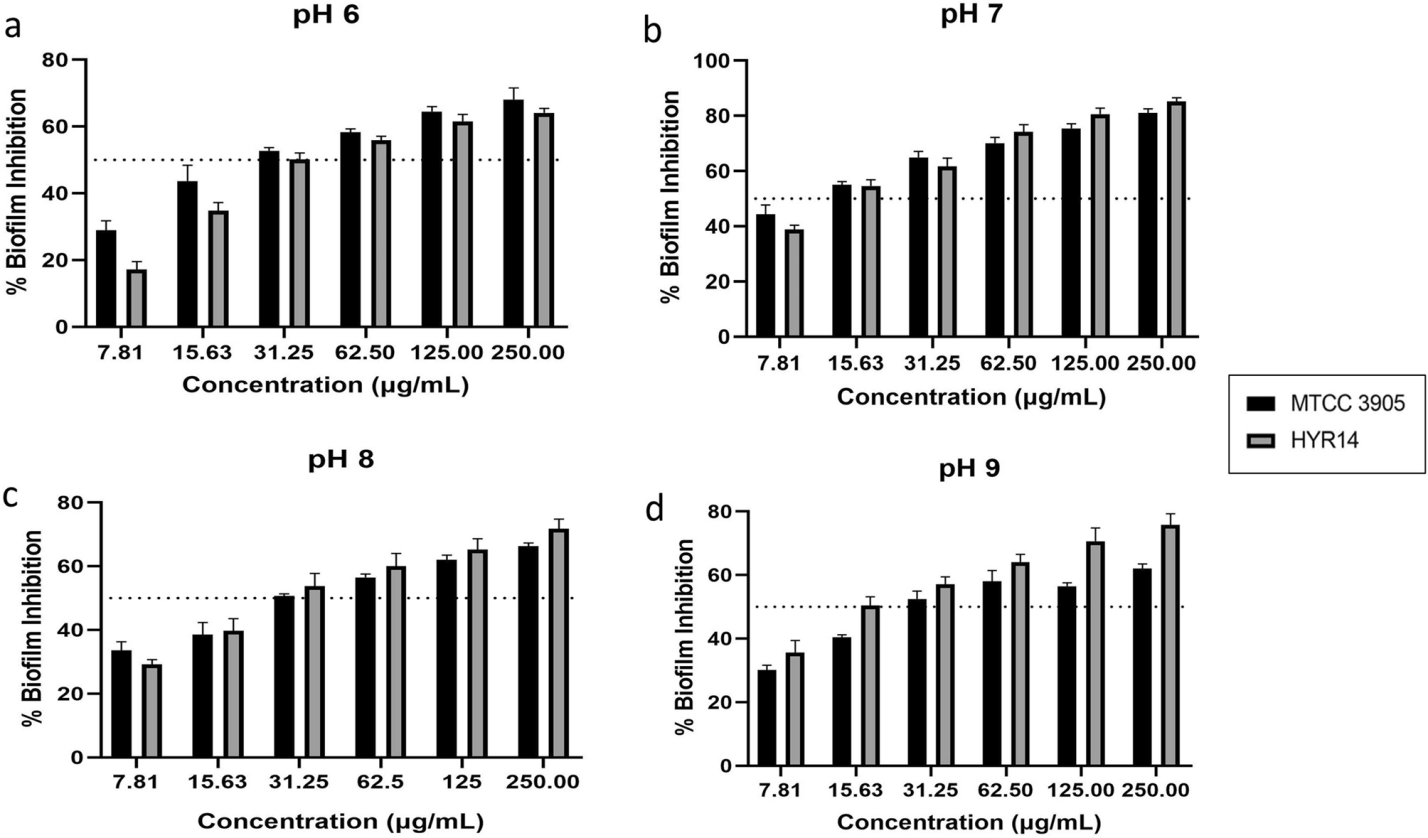
Percentage inhibition of *V. cholerae* MTCC 3905 and *V. cholerae* HYR14 biofilm formation at (**a**) pH 6, (**b**) pH 7, (**c**) pH 8, and (**d**) pH 9 after exposure to various concentrations of cellulose acetate phthalate nano-formulation of 2M4VP (CAP-2M4VP) for 24 h at 37 °C. At the concentration of 31.25 μg/mL, CAP-2M4VP inhibits about 50% of biofilm formation by *V. cholerae* MTCC 3905 and HYR14 at pH 6, pH 7, pH 8 and pH 9.

**Figure 9:**
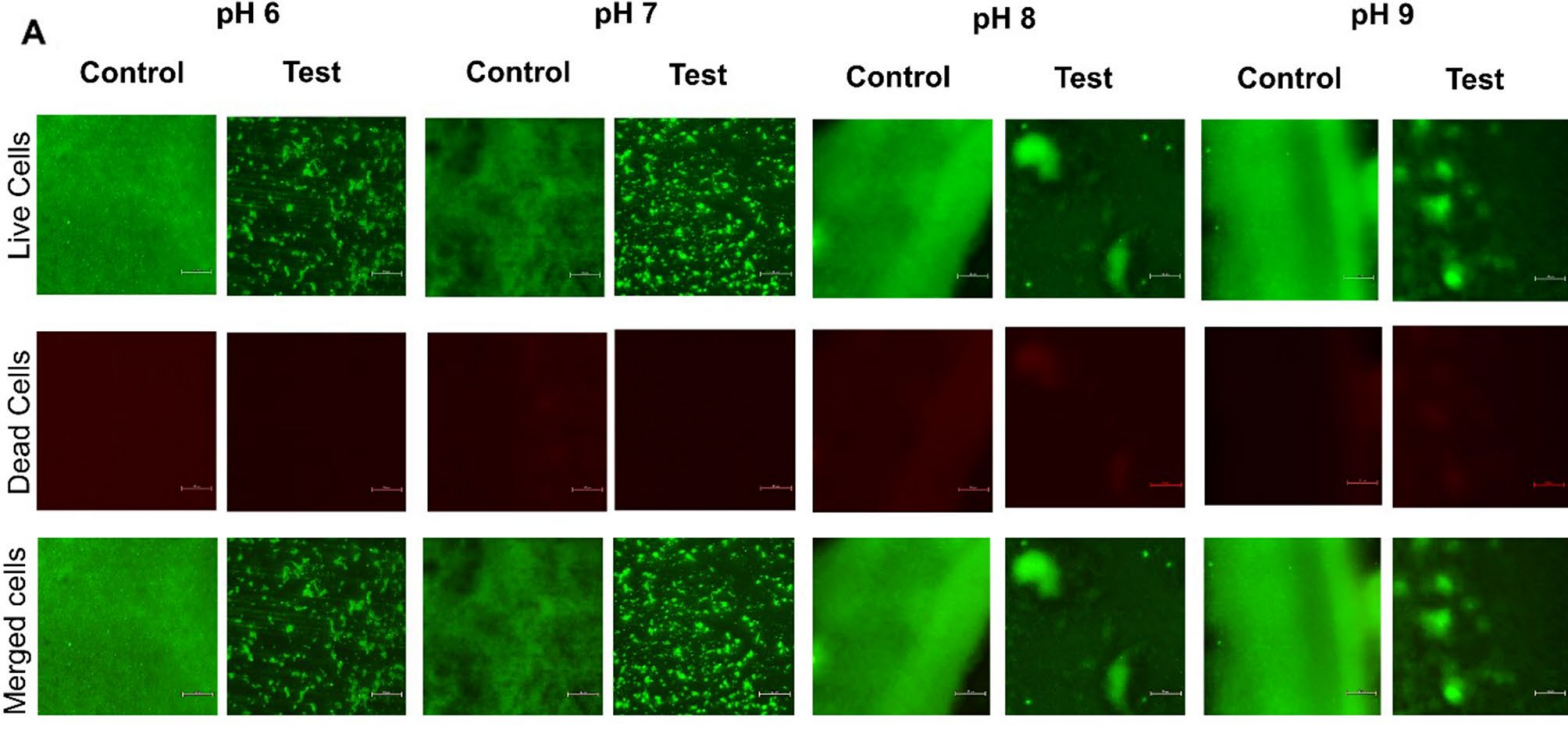
Fluorescent microscopy images of the biofilm formed by *V. cholerae* MTCC 3905 in the CAP-2M4VP treated (Test) and untreated (Control) conditions incubated statically for 24 h at 37 °C. At pH 6, pH 7, pH 8 and pH 9, the biofilms formed by *V. cholerae* MTCC 3905 upon treated with 31.25 μg/mL of CAP-2M4VP nano-formulation was minimal compared to the biofilm formed in the untreated (control) condition.

**Figure 10:**
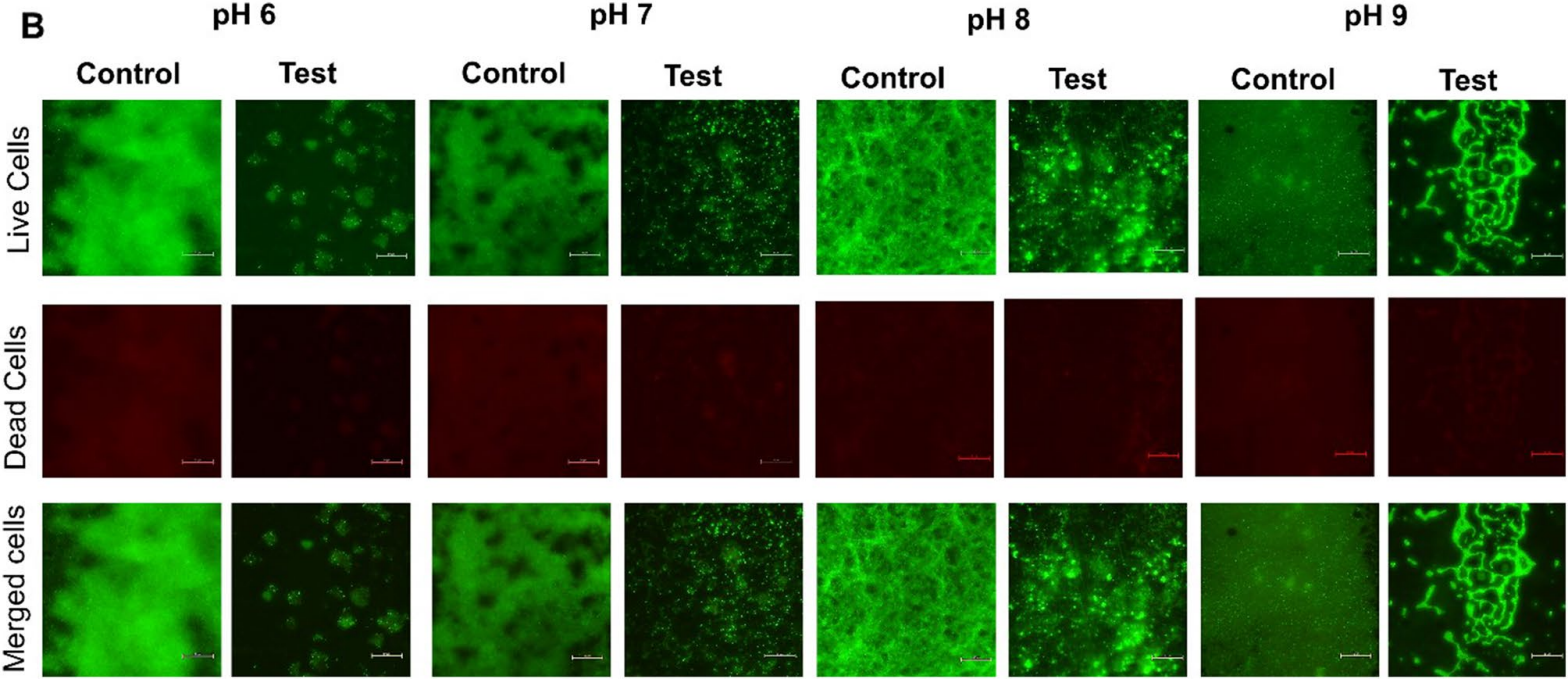
Fluorescent microscopy images of the biofilm formed by *V. cholerae* HYR14 in the CAP-2M4VP treated (Test) and untreated (Control) conditions incubated statically for 24 h at 37 °C. At pH 6, pH 7, pH 8 and pH 9, the biofilms formed by *V. cholerae* HYR14 upon treated with 31.25 μg/mL of CAP-2M4VP nano-formulation was minimal compared to the biofilm formed at the untreated (control) condition.

At various concentrations tested, it was observed that CAP-2M4VP did not affect the growth of *V. cholerae* MTCC 3905 and HYR14 strains. Even at a higher concentration of 500 μg/mL, the growth of both the strains were not affected significantly. This indicated that the CAP-2M4VP nanoparticles were non-bactericidal (Supplementary Figs. S3–S6). The CAP-2M4VP nanoparticles displayed an ability to inhibit the biofilm formation of *V. cholerae* MTCC 3905 and HYR14 strains at pH 6, pH 7, pH 8, and pH 9, dose-dependently. The minimum concentration of CAP-2M4VP that inhibited about 50% of *V. cholerae* MTCC 3905 biofilm formation was 31. 25 μg/mL at pH 6, 8 and 9, and 15.63 μg/mL at pH 7. Similarly, the minimum concentration of CAP-2M4VP that inhibited 50% of *V. cholerae* HYR14 biofilm was 31. 25 μg/mL at pH 6, and 8 and 15.63 μg/mL at pH 7 and 9. Overall, at 31.25 μg/mL CAP-2M4VP could effectively inhibit about 50% of biofilm formation by *V. cholerae* MTCC 3905 and HYR14 at pH 6, pH 7, pH 8 and pH 9. Highest level of biofilm inhibition (about 80%) was noted at the concentration of 250 μg/mL, for both MTCC 3905 and HYR14 strains at pH 7 (Fig. 8a–d). Fluorescent microscopy images revealed that untreated *V. cholerae* strains formed condensed and compact biofilm. When treated with CAP-2M4VP nanoparticles (31.25 μg/mL) the biofilm formed by *V. cholerae* MTCC 3905 and HYR14 were minimal and disintegrated (Figs. 9, 10). This shows that the controlled release of CAP-2M4VP at intestinal pH can potentially inhibit the formation of *V. cholerae* biofilm.

### Reduction of *V. cholerae* cell adherence

**Figure 11:**
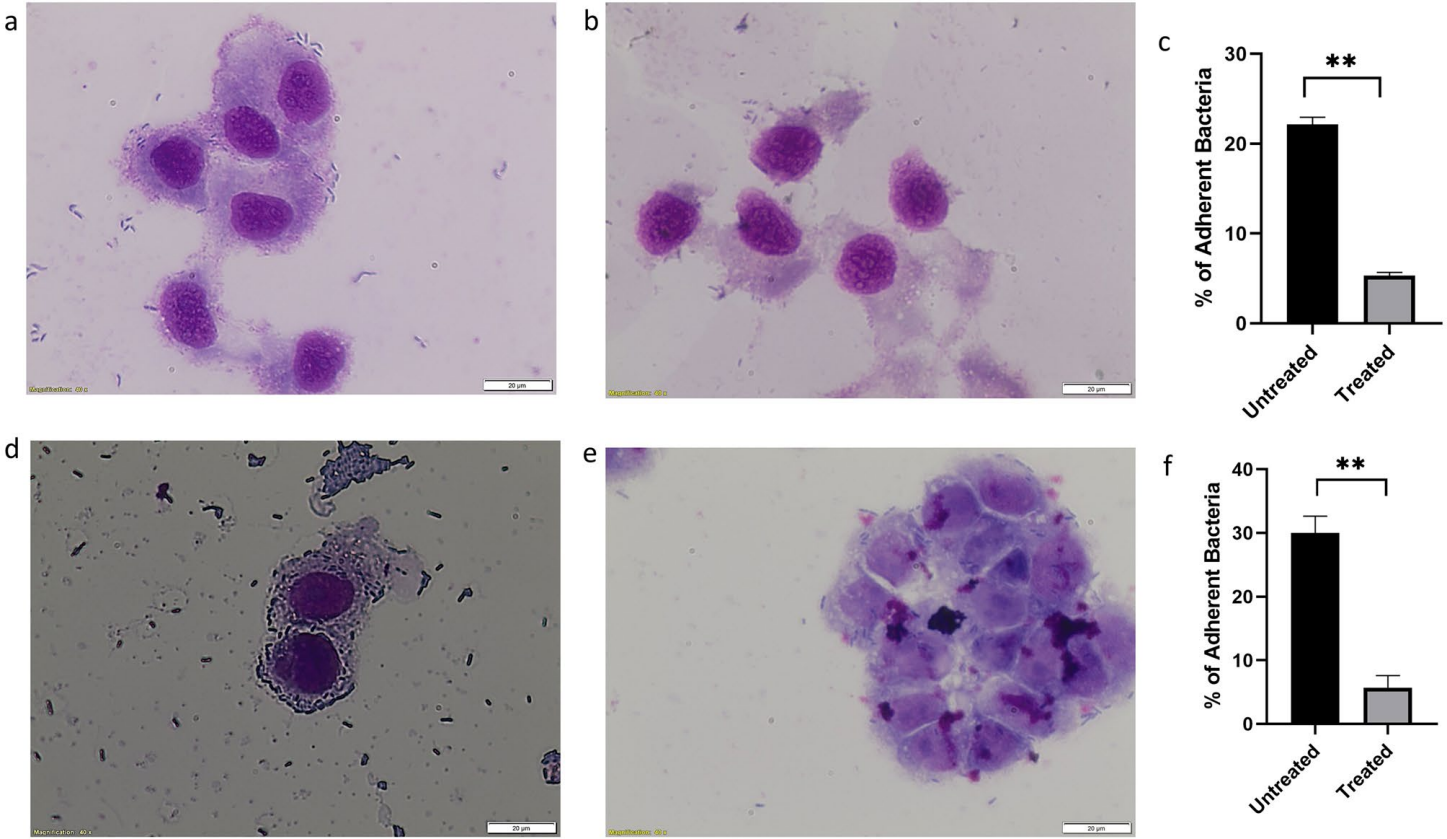
Adhesion of *V. cholerae* cells to Int 407 cell lines. (**a**) Light microscopy image showed the adhesion of *V. cholerae* MTCC 3905 to untreated Int 407 cell lines. (**b**) Light microscopy image of the adhesion of *V. cholerae* MTCC 3905 to Int 407 cell lines treated with CAP-2M4VP nano-formulation. (**c**) Percentage of adherent *V. cholerae* MTCC 3905 cells to Int 407 cell lines. (**d**) Light microscopy image of the adhesion of *V. cholerae* HYR14 to untreated Int 407 cell lines. (**e**) Light microscopy image of the adhesion of *V. cholerae* HYR14 to Int 407 cell lines treated with CAP-2M4VP nano-formulation. (**f**) Percentage of adherent *V. cholerae* HYR14 to Int 407 cells. Student *t*-test was used for statistical significance analysis. **Indicates significant difference (**p < 0.01) compared to the untreated.

When compared to the adhesion of *V. cholerae* strains to Int 407 cell line under untreated condition, the adhesion of *V. cholerae* strains to CAP-2M4VP treated Int 407 cell lines were minimal. Light microscopy images of Int 407 cell lines infected with *V. cholerae* MTCC 3905 (Fig. 11a,b) and HYR14 strains (Fig. 11d,e) showed a reduction in bacterial cell adherence. Enumeration of *V. cholerae* MTCC 3905 and HYR14 adhered to Int 407 cell lines revealed that upon treated with CAP-2M4VP (31.25 μg/mL), the percentage of *V. cholerae* cells attached to Int 407 cell lines were significantly less compared to the untreated Int 407 cells (Fig. 11c,f). Adherence is a key factor in *V. cholerae* pathogenesis as this leads to effective colonization of the small intestine which in turn allows cholera toxin to reach the epithelium causing cholera^28^. Hence, the display of reduction of adherence of *V. cholerae* strains to Int 407 cell lines indicates the potential of CAP-2M4VP to affect the initial stage of infection.

### Downregulation of virulence by CAP-2M4VP

**Figure 12:**
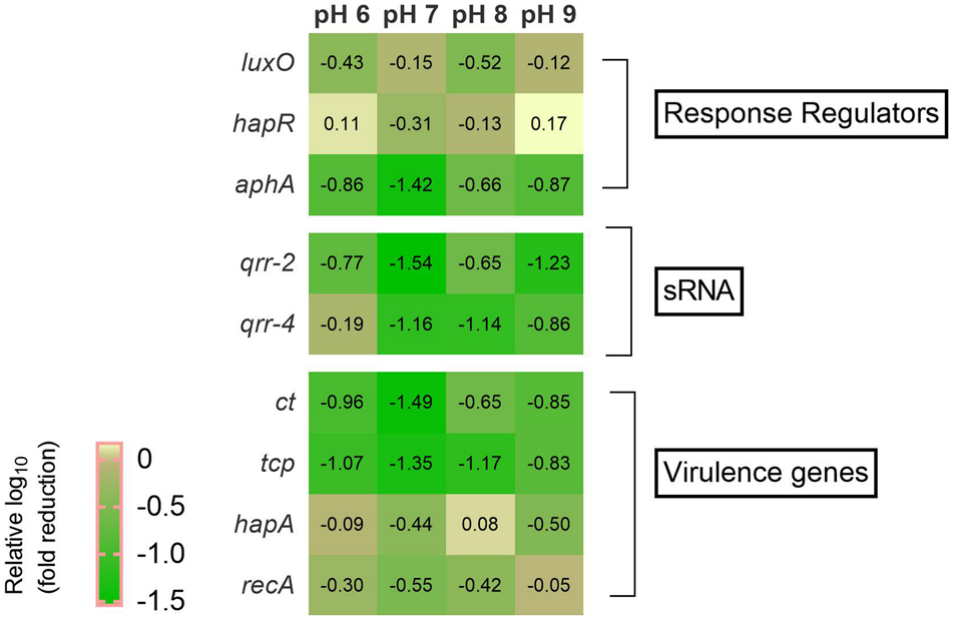
Heat map representation of the relative log_10_-fold downregulation of the expression of genes encoding quorum sensing response regulators, cholera toxin, and toxin co-regulated pili in *V. cholerae* MTCC 3905 by CAP-2M4VP at pH 6, pH 7, pH 8, and pH 9.

Gene regulation analysis by qRT-PCR revealed that CAP-2M4VP at all pHs downregulates the expression of various virulence encoding genes in MTCC 3905. At pH 6, *tcp* was downregulated by 1 log_10_ fold indicating the reduction in the expression of toxin co-regulated pili. At pH 7, 1 log_10_ reduction in the expression of *aphA*, *qrr 2, qrr-4, tcp,* and *ct* was noted. At pH 8, 1 log_10_ reduction in *qrr-4* and *tcp* and at pH 9, 1 log_10_ reduction in *qrr-2* was observed. Overall, at pH 6, pH 7, pH 8, and pH 9, downregulation in the expression of genes encoding the response regulator *aphA*, sRNA *qrr 2, and qrr-4,* virulence factors including toxin co-regulated pili (TCP) and cholera toxin (CT) were observed (Fig. 12).

## Discussion

In our previous study, *in-silico* analysis revealed that 2-methoxy-4-vinylphenol present in the organic extract of *Micromonospora* sp. RMA46 could interact with the active site of LuxO of *V. cholerae* potentially inhibiting the activity of LuxO^11^. *V. cholerae* has developed a sophisticated acid tolerance response enabling it to survive the gastric acids in the human stomach^29^. Upon escaping the acid barrier, the bacterial cells reach the human small intestine, which is the primary site of infection^30^. To facilitate the controlled release of 2M4VP in the small intestinal condition, a pH-dependent polymer cellulose acetate phthalate was used for the preparation of polymeric nanoparticles of 2M4VP. This preparation was analyzed in-vitro for effective controlled drug release and, its potential to attenuate virulence in *V. cholerae.*

The smaller size of the CAP-2M4VP nanoparticles indicated the compatibility of the emulsion evaporation method in the formulation of nanoparticles. The formation of nanoparticles less than 100 nm has been beneficial in terms of gastrointestinal drug delivery. Smaller-sized drug nanoparticles allows increased drug accumulation at the site of infection, improved transport, and retention in the gastrointestinal tract, uniform drug distribution, and increased drug uptake in cells and tissues in the gastrointestinal tract^31^. The zeta potential of the CAP-2M4VP was net negative charge and the polydispersity index was 0.251 ± 0.07 indicating a narrow size distribution of the CAP-2M4VP nanoparticle^32,33^.

Good stability of the nanoparticles prevents the release of the drug during storage, reducing the administrative concentration of the drug to induce effective therapeutic activity^34^. Also, nanoparticles with a negative charge have been suggested to have a greater cycle time in-vivo than positively charged nanoparticles^35^. As the mucus is negatively charged, the negative charge of CAP-2M4VP nanoparticles could be beneficial for mucus penetration due to the absence of ionic interaction between the CAP-2M4VP and mucus^36^.

SEM analysis has revealed a transformation of the irregular flakes of the pure drug 2M4VP to more regular spherical-shaped CAP-2M4VP nanoparticles indicating uniformity in the formulation. The FTIR analysis has shown that no functional group has been added or removed from the CAP-2M4VP signifying no loss of functionality of the 2M4VP. XRD displayed the solid-state transition of the 2M4VP from crystalline to an amorphous pattern in CAP-2M4VP. The amorphous XRD pattern is characteristic of cellulose acetate phthalate^27^. The loss of crystalline peaks in XRD analysis by CAP-2M4VP shows that the drug 2M4VP has effectively encapsulated within CAP. Other advantages of amorphous nanoparticle formulation is an increase in the dissolution and bioavailability^16,27,37^.

The complete release of the 2M4VP from CAP nano-formulation was observed at pH 7.0 at 40th h. However, a burst release of about 51% was observed at pH 7.0, indicating a release triggered by diffusion and release of drugs near the surface of the nanoparticles. The slow linear release noted after that could have been caused by the diffusion of 2M4VP from the bulk of the nanoparticle^38^. Controlled drug releases were observed in all media of various pH included in this study, indicating the possibility of achieving a targeted release of drug 2M4VP in a controlled manner. When fitting into different mathematical kinetics models, the cumulative percentage of drug release in-vitro revealed that the release of 2M4VP from the CAP nanoparticles was concentration gradient-based diffusion and followed Fickian law as the n values (release coefficient) was < 0.45 in all the pHs.

The small intestine of humans has a large surface area of ~ 400 m^2^. Once subduing the acidic pH in the stomach, the drug reaching the small intestine faces many physiological barriers, including enzymes, bile, and mucus. The mucus especially could capture and eliminate foreign bodies, which may be beneficial in protecting against gastrointestinal pathogens but could be disadvantageous by limiting drug availability in the small intestine^39,40^. Hence, an orally administrated drug must demonstrate efficient permeability across the mucus and reach the epithelium-a site where *V. cholerae* adheres and secretes virulence factors for infection^41,42^. Here, the CAP-2M4VP demonstrates a tenfold increased permeability across the intestinal membrane than the pure drug 2M4VP indicating that the polymeric nano-formulation of 2M4VP with CAP could have increased mucus permeability^43^.

Few studies have reported the anti-inflammatory activity of 2M4VP^44,45^. In these studies, various concentrations of 2M4VP demonstrated different levels of cytotoxicity against different cell lines. 2M4VP was reported to have cytotoxicity against Panc-1, while non-cytotoxic to SNU-213 and 293 T cell lines^45^. At concentrations lesser than 40 μM, 2M4VP was non-cytotoxic to RAW264.7 cells. Under LPS-induced conditions, 2M4VP inhibits nitric oxide, prostaglandins, inducible NO synthase, and cyclooxygenase-2^44^. In this study, the CAP-2M4VP at concentrations between 50 and 6.25 μg/mL was non-cytotoxic to Int 407 cell lines indicating the non-toxicity of the nano-formulation at lower concentrations.

Anti-virulence agents supposedly disarm key virulence determinants of a pathogen affecting its infectivity rather than viability (growth). Such agents have an insignificant effect on bacterial cell fitness and do not force bacteria to develop resistance^46,47^. Even at a higher concentration (500 μg/mL), CAP-2M4VP did not express growth inhibitory properties against *V. cholerae* reference strain (MTCC 3905) and multidrug resistant (MDR) clinical strain (HYR14) at pH 6, 7, 8, and 9. This indicates the non-bactericidal property of the CAP-2M4VP nano-formulation and that the drug targets the QS system and not the growth of the planktonic cell.

As *V. cholerae* cells are sensitive to gastric acids in the stomach, the biofilm phenotype of *V. cholerae* protects the cells from acid shock. This enables the cells to evade the acidic pH in the stomach and, upon reaching the small intestine, enhances their colonization at their site of infection. Indeed, the acid tolerance response (ATR) has been identified as the contributing factor for increased colonization of *V. cholerae* in-vivo^48,49^. Mouse model studies showed that *V. cholerae* biofilm architecture facilitates virulence. Intact and dispersed *V. cholerae* biofilm supports more significant colonization outcompeting planktonic cell colonization in-vivo. And the infectious dosage of biofilm cells was also lesser than the planktonic cells^50^. In this study, at 31.25 μg/mL, about 50% inhibition of biofilm formation was noted in both reference and MDR clinical strains at pH 6, 7, 8, and 9. Moreover, fluorescent microscopy images revealed that the treatment of CAP-2M4VP led to the absence of well-established biofilms at all pHs. The biofilm formed by *V. cholerae* cells in the presence of CAP-2M4VP is thinner and disintegrated at pH 6, 7, 8, and 9. As the biofilm structure is crucial for survival against acidic shock and colonisation^51^, failure to form a mature well-structured biofilm by *V. cholerae* could affect its survival, intestinal colonization, and hypervirulence property.

Adherence is one of the critical variables affecting *V. cholerae* colonization in the small intestine. *V. cholerae* must penetrate the thick mucus barrier in the small intestine using mucinases^52,53^, followed by reversibly and irreversibly attachment to the epithelial cells to first identify a suitable site for infection^54,55^. Various adhesion molecules, including GbpA (GlcNAc-binding protein), facilitates *V. cholerae* adhesion, and a decrease in the adherence of *V. cholerae* to epithelium has directly affected the fitness of colonisation^28^. In this study, CAP-2M4VP reduces the adherence of *V. cholerae* to Int 407 cell lines in-vitro suggesting that CAP-2M4VP could also affect the colonization of *V. cholerae*, an indispensable stage of *V. cholerae* life cycle.

Parallel QS circuits function co-ordinately to regulate the expression of virulence in *V. cholerae*, including the production of biofilm, protease, and cholera toxin through response regulators LuxO and HapR^56^. At low cell density, the phosphorylated LuxO in its active state as a kinase represses *hapR* to positively regulate the genes encoding biofilm formation and cholera toxin production^7^. Gene expression analysis in the presence of CAP-2M4VP at pH 6, 7, 8, and 9 showed reductions in the expression of genes encoding virulence in different pHs. Overall, in all the pHs the expression of *tcp* is highly reduced, followed by sRNA *qrr-2* and *qrr-4, ct,* and *aphA.* The toxin-coregulated pili (TCP) is a type IV pili and vital for the attachment of *V. cholerae* to enterocytes and M cells^57,58^. TCP also increases the production of MUC4 mucin, which facilitates *V. cholerae* adhesion right from the initial stages of infections^59^. Here, the notable decrease in the expression of *tcp* across the pHs indicates that CAP-2M4VP could potentially affect the adherence of *V. cholerae* in the small intestine. Similarly, the reduction in the expression of *ct,* a gene encoding the production of cholera toxin, is also observed across all pHs. The QS signal molecules CAI-1 and AI-2 converge at LuxO, which in association with σ^N^ positively regulates the expression of sRNA (*qrr 1–4*). The sRNAs activate the transcription of *aphA,* which transcribes the virulence genes *tcp* and *ct.* The reduction in the expression of genes encoding *qrr2*, *qrr4*, *aphA*, *tcp*, and *ct* indicates downregulation in the expression of all these genes could be possible through the inactivation of LuxO.

To conclude, the polymeric nano-formulation of 2M4VP encapsulated in cellulose acetate phthalate was evaluated for its potential to inhibit the virulence of *V. cholerae*. The characterization of polymeric nano-formulation of 2M4VP indicated mono-dispersion and good size distribution. FTIR, XRD, and TGA–DSC analysis showed that chemical interaction between the polymer and the drug was absent, solid-state conversion from the crystalline shape of the drug to amorphous and increase in thermal stability. This indicated polymeric nano-formulation has introduced many biocompatible properties to 2M4VP. In addition to this, the controlled drug release pattern and increase in permeation across the intestinal membrane ex-vivo further emphasize the bio-effectivity of the formulation of 2M4VP in CAP. Furthermore, the formulation is non-bactericidal, non-cytotoxic, inhibits biofilm formation, and adherence, and downregulates the major virulence encoding genes in *V. cholerae*.

## Supplementary Information

Supplementary Information.

## Data Availability

The datasets generated during and/or analysed during the current study are available from the corresponding author on reasonable request.
