# Supplementary material for: Development of a smart pH-responsive nano-polymer drug, 2-methoxy-4-vinylphenol conjugate against the intestinal pathogen, Vibrio cholerae
Source: Sci Rep. 2023 Jan 23;13:1250. doi: 10.1038/s41598-023-28033-0 (PMC9871008; doi:10.1038/s41598-023-28033-0)
Supplement: Supplementary file 1 — Supplementary Information. [file 41598_2023_28033_MOESM1_ESM.docx]

**Development of a smart pH-responsive nano-polymer drug, 2-methoxy-4-vinylphenol conjugate against the intestinal pathogen, *Vibrio cholerae***

Hema Bhagavathi Sarveswari^1^, Krishna Kant Gupta^2^, Ramyadevi Durai^3*^ and Adline Princy Solomon^1*^

*^1^ Quorum Sensing Laboratory, Centre for Research in Infectious Diseases (CRID), School of Chemical and Biotechnology, SASTRA Deemed to be University, Thanjavur, 613401, India.*

*^2^ School of Chemical and Biotechnology, SASTRA Deemed to be University, Thanjavur, 613401, India.*

*^3^ Pharmaceutical Technology Laboratory, School of Chemical and Biotechnology, SASTRA Deemed to be University, Thanjavur, 613401, India.*

**Supplementary File**

**Supplementary Figure 1.** Schematic representation of the preparation of CAP-2M4VP nanoparticle


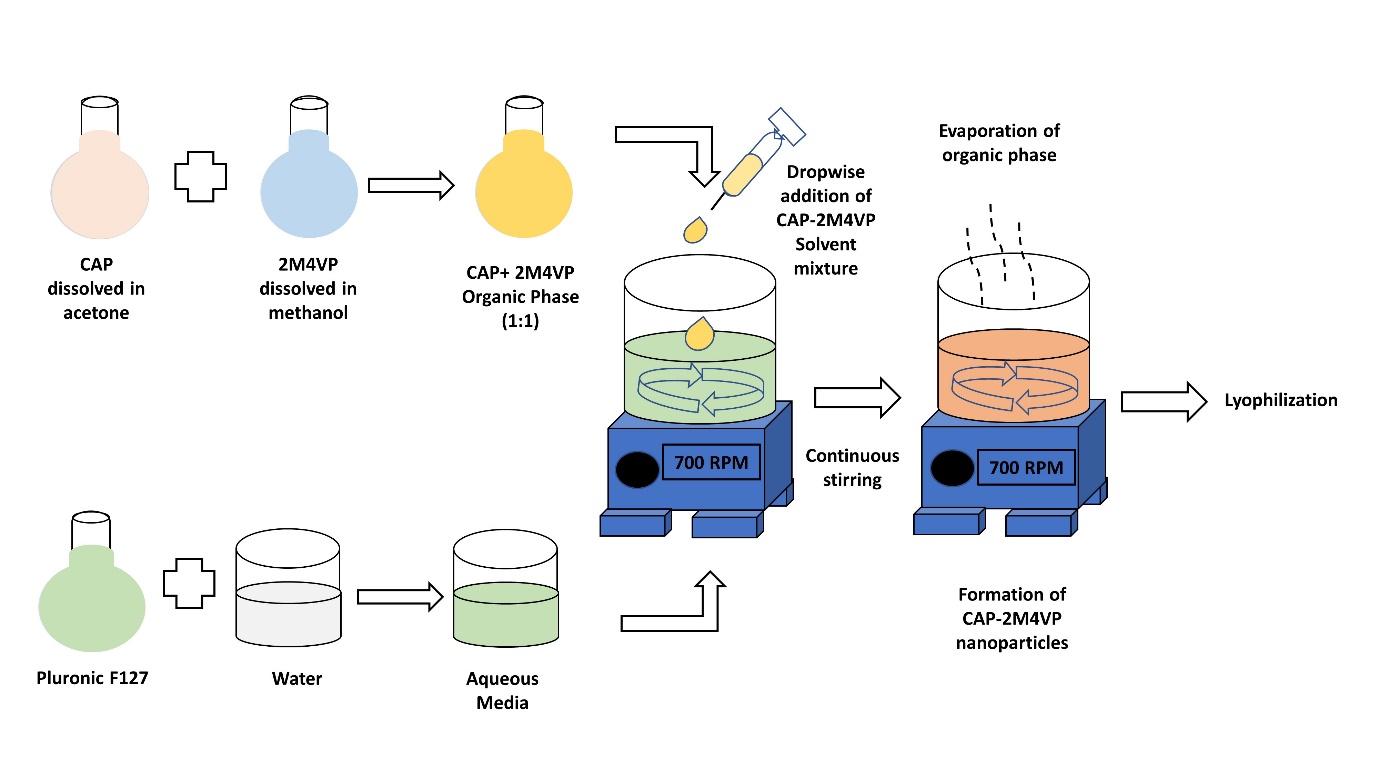


**Supplementary Figure 2.**  Particle size (A) and zeta potential (B) of polymeric nanoformulation of 2M4VP (CAP-2M4VP).


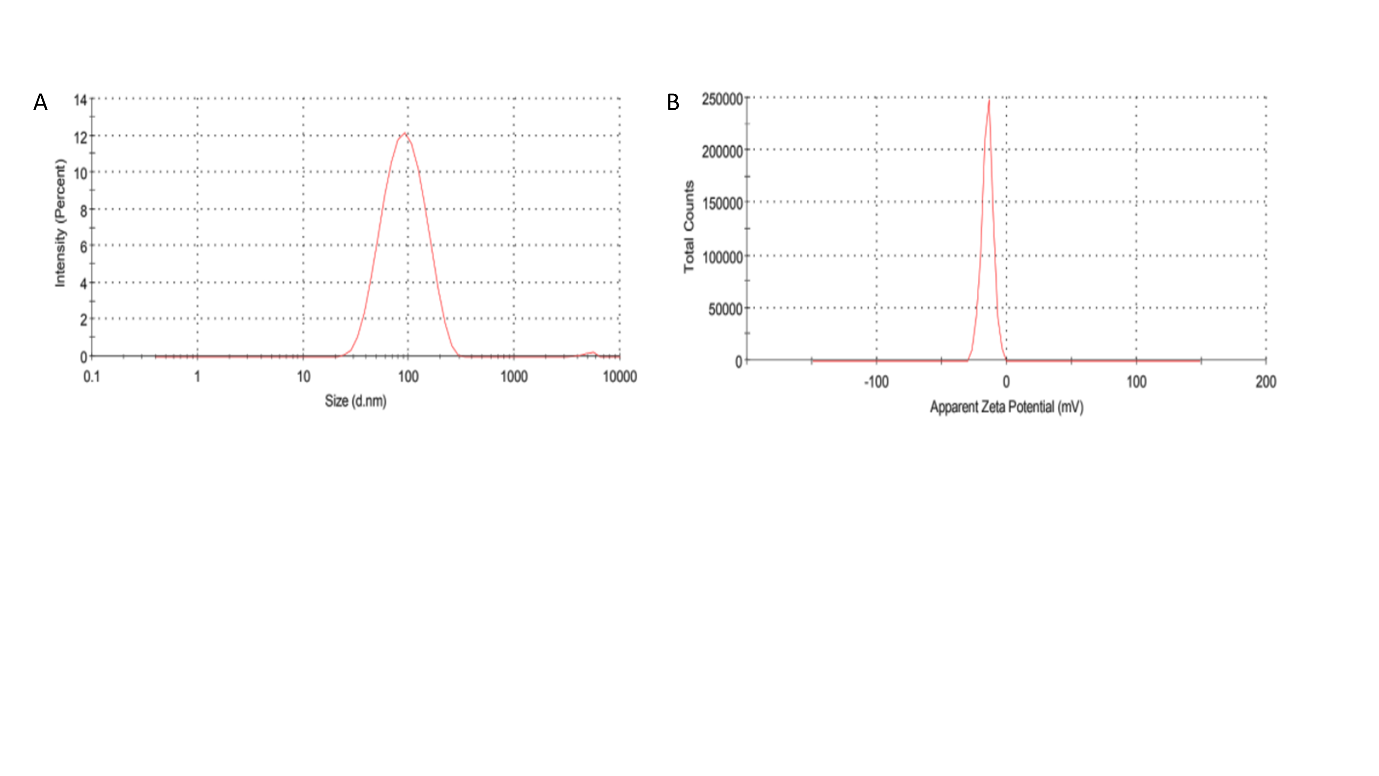


**Supplementary Figure 3.**  Effect of CAP-2M4VP on the growth of *V. cholerae* MTCC 3905 and HYR14 after 24 hours at pH 6.

**
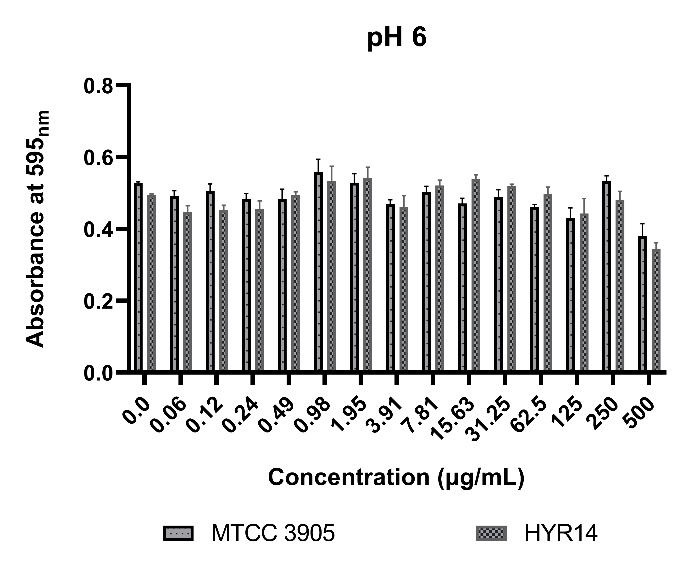
**

**
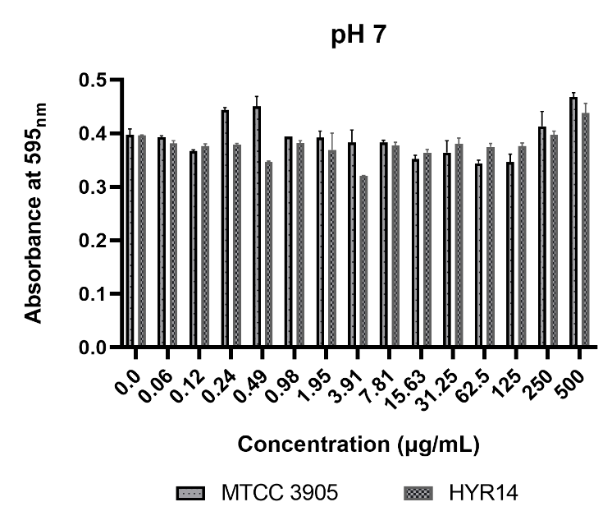
Supplementary Figure 4.** Effect of CAP-2M4VP on the growth of *V. cholerae* MTCC 3905 and HYR14 after 24 hours at pH 7.

**Supplementary** **
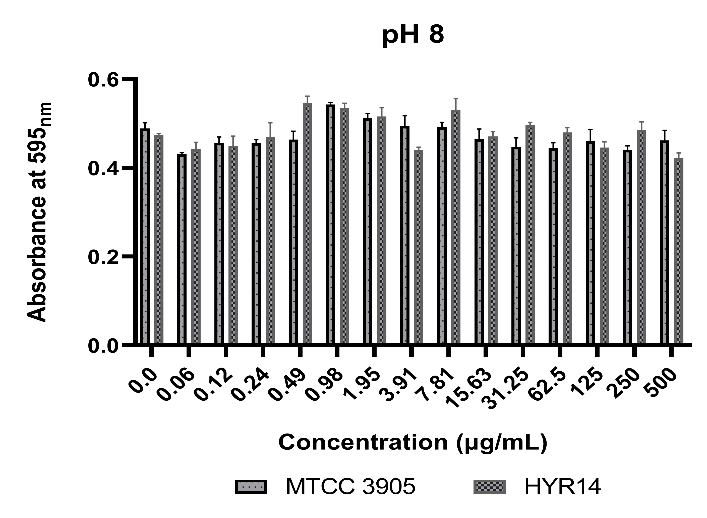
Figure 5.** Effect of CAP-2M4VP on the growth of *V. cholerae* MTCC 3905 and HYR14 after 24 hours at pH 8.

**Supplementary Figure 6.** Effect of CAP-2M4VP on the growth of *V. cholerae* MTCC 3905 and HYR14 after 24 hours at pH 9.

**
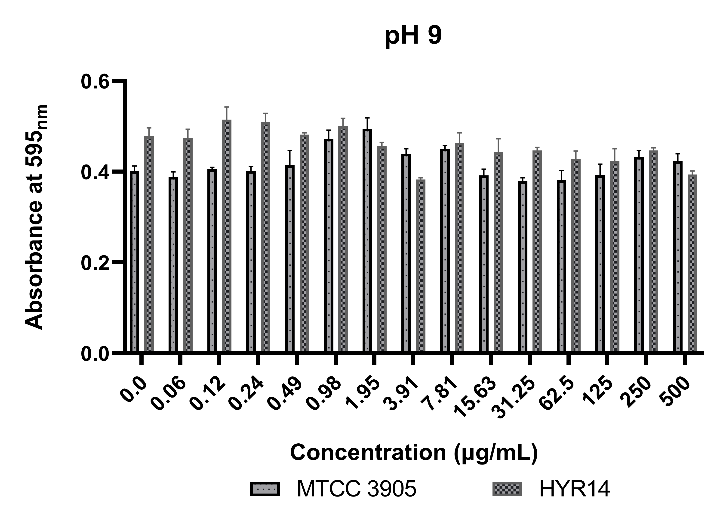
**

**Supplementary Table 1.** Primers used for gene expression analysis

| **Gene** | **Forward primer sequence**  **(5’ – 3’)** | **Reverse primer sequence**  **(3’ – 5’)** | **Reference** |
| --- | --- | --- | --- |
| *ct* | TATGCCAAGAGGACAGAGTGAG | AACATATCCATCATCGTGCCTAAC | ^60^ |
| *tcp* | CGTTGGCGGTCAGTCTTG | CGGGCTTTCTTCTTGTTCG | ^60^ |
| *hapA* | ACGGTACAGTTGCCGAATGG | GCTGGCTTTCAATGTCAGGG | ^61^ |
| *hapR* | CCAACTTCTTGACCGATCAC | GGTGGAAACAAACAGTGGCC | ^62^ |
| *qrr-2* | GGTGACCCTTGTTAAGCCGA | CTATTCACTTCAACGTCAGTTGGC | ^26^ |
| *qrr-4* | TGACCCTTCTAAGCCGAGGG | GAACAATGGTGTTCACTTCAACG | ^26^ |
| *rec A* | ATTGAAGGCGAAATGGGCGATAG | TACACATACAGTTGGATTGCTTGAGG | ^63^ |
| *luxO* | GCGAAAGTGGTA CAGGTAAA | ATCAGATCTTTCGGAATGGC | ^63^ |

**Supplementary Table 2.** Release kinetics of CAP polymeric nano-formulation of 2M4VP (CAP-2M4VP).

| **Kinetics Model** | **Parameters** | **pH 1.2** | **pH 5.8** | **pH 7** | **pH 8** | **pH 9** |
| --- | --- | --- | --- | --- | --- | --- |
| **Zero order** | **R^2^** | 0.4503 | 1.2436 | 0.4118 | 0.4304 | 1.7858 |
|  | **K_0_** | 1.719 | 1.900 | 3.148 | 1.922 | 1.934 |
|  | **SS** | 6237.5447 | 9447.6506 | 20041.2991 | 7435.7775 | 11611.2526 |
| **First order** | **R^2^** | 0.0271 | 0.5434 | 0.6572 | 0.0768 | 0.9007 |
|  | **K_1_** | 0.026 | 0.031 | 0.121 | 0.031 | 0.033 |
|  | **SS** | 4184.1863 | 6499.3417 | 4866.4044 | 4799.2933 | 7922.0191 |
| **Higuchi** | **R^2^** | 0.7066 | 0.3813 | 0.7147 | 0.6958 | 0.1608 |
|  | **K_H_** | 9.457 | 10.531 | 17.267 | 10.531 | 10.831 |
|  | **SS** | 1261.7284 | 2605.5058 | 4050.4897 | 1581.5030 | 3497.7576 |
| **Korsemeyer-Peppas** | **R^2^** | 0.9905 | 0.9767 | 0.9900 | 0.9808 | 0.9712 |
|  | **K_KP_** | 19.779 | 26.327 | 35.978 | 22.210 | 29.033 |
|  | **n** | 0.261 | 0.202 | 0.262 | 0.258 | 0.179 |
|  | **SS** | 40.7947 | 98.3140 | 142.5851 | 99.9653 | 120.2230 |
| **Hixson-Crowell** | **R^2^** | 0.1172 | 0.7564 | 0.5100 | 0.0728 | 1.1741 |
|  | **K_HC_** | 0.008 | 0.009 | 0.032 | 0.009 | 0.009 |
|  | **SS** | 4804.8561 | 7396.2969 | 6955.8384 | 5577.0266 | 9061.8175 |
| **Hopfenberg** | **R^2^** | 0.0268 | 0.5439 | 0.6571 | 0.0762 | 0.9011 |
|  | **K_HB_** | 0.000 | 0.000 | 0.000 | 0.000 | 0.000 |
|  | **SS** | 4185.4535 | 6501.1905 | 4867.3656 | 4802.0359 | 7923.7080 |
| **Baker-Lonsdale** | **R^2^** | 0.7990 | 0.5445 | 0.9086 | 0.7928 | 0.3837 |
|  | **K_BL_** | 0.002 | 0.003 | 0.010 | 0.003 | 0.003 |
|  | **SS** | 864.6038 | 1917.9818 | 1297.7094 | 1077.3329 | 2568.6804 |
| **Weibull** | **R^2^** | 0.9943 | 0.9739 | 0.9707 | 0.9748 | 0.9944 |
|  | **SS** | 24.6371 | 110.0523 | 416.2617 | 131.1917 | 23.3906 |
| **Gompertz** | **R^2^** | 0.9893 | 0.9656 | 0.9345 | 0.9583 | 0.9831 |
|  | **SS** | 45.8105 | 144.9309 | 930.3335 | 216.7893 | 70.5727 |
